# Housework Reduces All-Cause and Cancer Mortality in Chinese Men

**DOI:** 10.1371/journal.pone.0061529

**Published:** 2013-05-07

**Authors:** Ruby Yu, Jason Leung, Jean Woo

**Affiliations:** 1 Department of Medicine and Therapeutics, The Chinese University of Hong Kong, Hong Kong, China; 2 Jockey Club Centre for Osteoporosis Care and Control, The Chinese University of Hong Kong, Hong Kong, China; Universität Bochum, Germany

## Abstract

**Background:**

Leisure time physical activity has been extensively studied. However, the health benefits of non-leisure time physical activity, particular those undertaken at home on all-cause and cancer mortality are limited, particularly among the elderly.

**Methods:**

We studied physical activity in relation to all-cause and cancer mortality in a cohort of 4,000 community-dwelling elderly aged 65 and older. Leisure time physical activity (sport/recreational activity and lawn work/yard care/gardening) and non-leisure time physical activity (housework, home repairs and caring for another person) were self-reported on the Physical Activity Scale for the Elderly. Subjects with heart diseases, stroke, cancer or diabetes at baseline were excluded (n = 1,133).

**Results:**

Among the 2,867 subjects with a mean age of 72 years at baseline, 452 died from all-cause and 185 died from cancer during the follow-up period (2001–2012). With the adjustment for age, education level and lifestyle factors, we found an inverse association between risk of all-cause mortality and heavy housework among men, with the adjusted hazard ratio (HR) of 0.72 (95%CI = 0.57–0.92). Further adjustment for BMI, frailty index, living arrangement, and leisure time activity did not change the result (HR = 0.71, 95%CI = 0.56–0.91). Among women, however, heavy housework was not associated with all-cause mortality. The risk of cancer mortality was significantly lower among men who participated in heavy housework (HR = 0.52, 95%CI = 0.35–0.78), whereas among women the risk was not significant. Men participated in light housework also were at lower risk of cancer mortality than were their counterparts, however, the association was not significant. Leisure time physical activity was not related to all-cause or cancer mortality in either men or women.

**Conclusion:**

Heavy housework is associated with reduced mortality and cancer deaths over a 9-year period. The underlying mechanism needs further study.

## Introduction

Regular physical activity has been documented to protect against many adverse health conditions, including non-communicable diseases such as coronary heart disease, type 2 diabetes, some cancers, hypertension, obesity, and depression [1]. The relationship between physical activity and all-cause mortality has also been extensively documented, and virtually all have indicated a reduction in risk [2]. In older populations among whom chronic diseases may be prevalent, promotion of physical activity is important. Regular physical activity such as exercising for at least 30 minutes per day on most days of the week has been recommended for adults aged 65 and above [1]. Some have demonstrated that even for those who did not meet the recommended levels, engaging in some physical activities may increase longevity [3]–[6]. Despite widely publicized recommendations, available data suggest that a large proportion of the general population remains physically inactive [7]. In Hong Kong, less than 30% of adults and the elderly were categorized as having sufficient physical activity (accumulation of ≥150 minutes of moderate or above intensity physical activity in a week) [8].

Older people were less likely to participate in moderate or vigorous recreational exercise, but non-leisure time physical activity, especially household related activity, comprises the majority of physical activity and may substitute for other types of activities [9]–[12]. In a survey of elderly British people, the percentage of performing indoor activities was 86%, of which 95% comprised housework [9]. Data from the National Human Activity Pattern Survey (NHAPS) of over 7,000 subjects revealed that household related activity contributed 35.2% of total energy expenditure compared to 5.2% of leisure time physical activity in subjects aged 65–74 years [11]. Another well conducted study has found that the prevalence of individuals achieving the recommended levels of physical activity increased by over threefold when household activities were included in the assessment [10]. In a population-based cross-sectional study in urban Chinese women aged 40–70 years, more than 90% of participants reported physical activity energy expenditure was from non-exercise activities [12]. However, little research has been devoted to studying the relation between household related activity and mortality, particularly among older people. Of the few published studies, some have demonstrated an inverse relation [13]–[17], whereas other has shown little association [18].

Using a data set from a health survey of 4,000 people aged 65 years and over living in all regions of Hong Kong, we examined the prevalence of levels of different types of physical activity, and their association with all-cause and cancer mortality over nine years of follow-up.

## Materials and Methods

### Subjects

Four thousand community-dwelling Chinese men and women aged 65 and older were recruited for a cohort study on osteoporosis and general health in Hong Kong between August 2001 and December 2003 by placing recruitment notices in community centers for older adults and housing estates. Several talks were also given at these centers explaining the purpose, procedures, and investigations to be carried out. Subjects were volunteers, and the aim was to recruit a stratified sample so that approximately 33% would each be aged 65 to 69, 70 to 74, and 75 and older. Those who were unable to walk independently, had had bilateral hip replacement, were not competent to give informed consent were excluded. Eligible subjects were invited to attend a health check at the School of Public Health, The Chinese University of Hong Kong. A team of trained research assistants admonished the study questionnaire and physical measurements for each subject on the same day. All subjects gave written consent and the study was approved by the clinical research ethics committee of the Chinese University of Hong Kong.

### Self-report Physical Activity

Physical activity level was assessed using the Physical Activity Scale of the Elderly (PASE). This is a 12-item scale measuring the average number of hours per day spent in leisure, household and occupational physical activities over the previous 7-day period. Subjects were asked to indicate how often (i.e., seldom, 1 to 2 days; sometimes, 3 to 4 days; or often, 5 to 7days) in the previous week they had engaged in a variety of activities. These included walking; activities of light (e.g., stretching, bowling, golf (with a cart), fishing at the pier or on a boat, Tai Chi, Qigong, ping pong or other similar activities), moderate (e.g., pairs tennis, badminton, social dance, golf (without cart), carry heaving things while walking on flat grounds (less than 5 kg) or other similar activities), or strenuous intensity (e.g., jogging, swimming, cycling, single tennis, aerobic exercise, hiking with a backpack, squash, basketball, exercise bike, rowing, carry heavy things up the stairs (e.g., a 5 kg pack of rice) or other similar activities); and muscle-conditioning activities (e.g., lifting weight, push up or other similar activities). Duration spent in these activities was categorized as less than 1 hour, 1 to 2 hours, 2 to 4 hours, or more than 4 hours per day. Furthermore, engagement in household activities such as light housework (e.g., dusting, washing dishes, hand wash clothes, ironing, hanging up wet clothes, cooking or buying groceries), heavy housework (e.g., vacuuming, scrubbing floors, mopping floors, washing windows, washing cars, moving furniture or moving portable gas cylinders), home repairs (e.g., painting, wall papering, electrical work), lawn work or yard care (e.g., lawn mowing, leaves removal, wood chopping), outdoor gardening, and caring for another person (e.g., children, dependent spouse or another adult) was categorized as yes or no. Time spent in paid or volunteer work involving at least some standing or walking was also recorded in total hours per week. A summary score of all the items reflect the daily physical activity level [19].

### Mortality

Mortality was confirmed by annual reports from the Hong Kong Death Registry. The cutoff date for determining mortality was March 31, 2012. Cancer causes of death was identified by the cause of death reported on the death certificate, and classified according to the International classification of Disease (ICD) version 10 codes as those ranging from C00 to C97.

### Other Measurements

A questionnaire consisting of information on demographics, education level, self-rated socio-economic status, living arrangement, self-reported medical history, dietary intake, smoking and alcohol use was administered by trained interviewers. Self-rated socioeconomic status was assessed by asking subjects to place a mark on an upright ladder with ten rungs, with the top rung representing people who rated themselves as having the most money, the most education and the most respected jobs, and the bottom rung representing people at the other extreme (Hong Kong Ladder). The use of this self-rated measure had been discussed elsewhere [20], [21]. Dietary intake was assessed using a food frequency questionnaire. Total energy intake and dietary quality index (DQI) were calculated. Height and weight were measured on the same day that the questionnaire was administered. Height was measured using the Holtain Harpenden stadiometer (Holtain Ltd, Crosswell, UK). Body weight was measured with participants wearing a light gown using the Physician Balance Beam Scale (Healthometer, Alsip, IL). BMI was calculated by dividing weight in kg by the square of height in meters. Frailty was quantified using the frailty index, constructed from a list of 47 binary variables, each representing the presence or absence of a deficit in physical, functional, psychological, nutritional and social domains, with a score of 1 representing a deficit of each variable. The maximum score is 47, and the frailty index was calculated by dividing the total score for each subject by 47 [22]–[25].

### Statistical Analysis

Statistical analyses were performed by using SPSS version 17.0 (IBM Corp, Somers, NY, USA). All analyses were done separately for men and women. Characteristics of decedents and survivors were compared. In this analysis, subjects who engaged in any activities of leisure time physical activity (either light, moderate, strenuous) at least 1 to 2 days per week were classified as part of the leisure time physical activity active group, and those who failed to meet this level as the among the leisure time physical activity inactive group. Since the number of subjects who engaged in muscle strength and endurance activities was small; those who engaged in muscle strength and endurance activities were merged into the group of subjects who engaged in strenuous activities. Similarly, the number of subjects who engaged in lawn work/yard care or outdoor gardening was small and thus merged together. All-cause and cancer mortality as on March 31, 2012 were analyzed using Cox proportional hazards regression. Person-time was calculated from enrollment until the date of death. Overall (by tertile) and different types of physical activities were entered into the models separately. Model 1 was adjusted for covariates that were relevant to mortality in older individuals including age (continuous), education level (no education, primary, secondary/matriculation, and university or above), socioeconomic status ladder (<5/≥5), total energy intake (kcal/day), DQI (continuous), smoking (never, past smoker, and current smoker), alcohol use (never, past drinker, and current drinker). Model 2 was adjusted for the factors in model 1 plus BMI (continuous), frailty index (continuous), living arrangement (live alone/live with spouse/others) and levels of leisure time physical activity/housework (continuous). Level of leisure time physical activity was adjusted in the models for the relationship between non-leisure time physical activity and all-cause mortality only while level of housework was adjusted in the models for the relationship between leisure time physical activity and all-cause mortality only. Subjects who did not engaged in any activities were used as the reference group. The proportional hazard assumption was confirmed by log minus log plots. All tests were two-sided, and a p value of <0.05 was taken as statistically significant.

## Results

**Figure 1:**
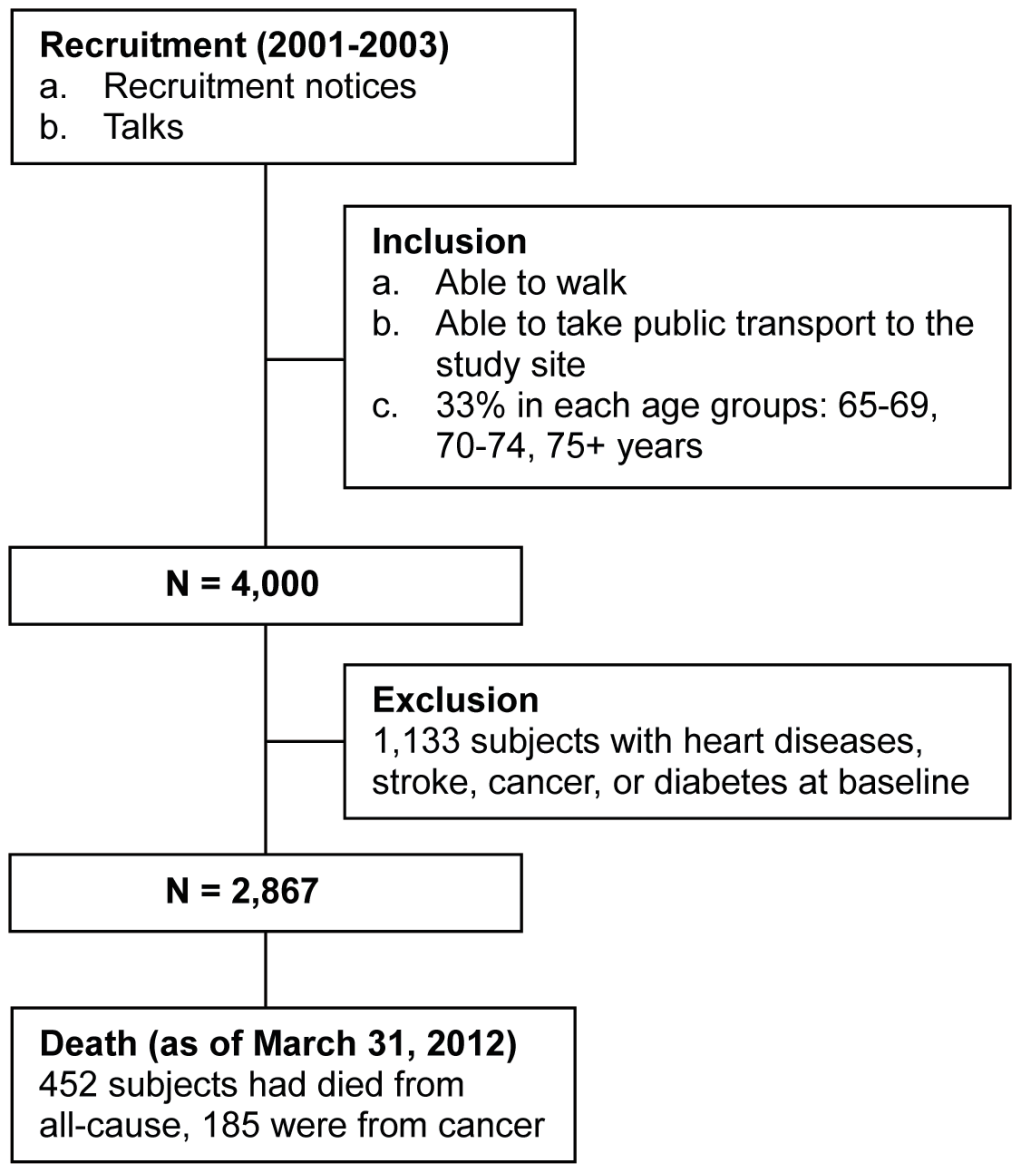
Recruitment flow chart.

After excluding subjects with diabetes, heart diseases, stroke, and cancer at baseline, the number of subjects was 2,867, with a mean age of 72 years at baseline. After a mean follow-up of 104.9±20.9 months (median 110.3 months), 452 (301 men and 151 women) had died from all-cause and 185 (123 men and 62 women) had died from cancer (Figure 1). Decedents in both men (n = 301) and women (n = 151) were older, were more likely be past smokers, had lower energy intake, lower DQI, and lower frailty index at baseline. In men, those who died also were less educated, more likely be past drinkers, had lower BMI, and were less active in participating moderate, strenuous, and non-leisure time activities. In women, no difference in any physical activity measures was observed between survivors and decedents (Table 1).

**Table 1:** Baseline characteristics of decedents and survivors.

	Men			Women		
Characteristic	Survivors (n = 1,116)	Decedents (n = 301)	P	Survivors (n = 1,299)	Decedents (n = 151)	P
Age, y	71.45±4.53	75.18±5.73	<0.0001	72.00±5.10	75.92±6.64	<0.0001
Education, %						
No education	5.3	5.3	0.018	37.0	37.7	0.066
Primary	53.8	63.1		44.9	52.3	
Secondary/matriculation	26.1	21.9		11.6	7.3	
University or above	14.9	9.6		6.5	2.6	
Social economic status ladder – Hong Kong, %						
<5	43.4	48.6	0.111	38.1	44.6	0.151
≥5	56.6	51.4		61.9	55.4	
Living arrangement, %						
Live alone	5.8	12.3	<0.0001	18.2	23.2	0.135
Live with spouse/others	94.2	87.7		81.8	76.8	
Smoking, %						
Never	39.8	25.2	<0.0001	91.8	78.8	<0.0001
Past smoker	47.2	59.8		6.2	16.6	
Current smoker	13.0	15.0		1.9	4.6	
Alcohol use, %						
Never	70.9	69.1	<0.0001	97.2	96.0	0.440
Past drinker	2.6	7.3		0.0	0.0	
Current drinker	26.5	23.6		2.8	4.0	
BMI, kg/m^2^	23.32±3.03	22.78±3.58	0.018	23.87±3.38	23.24±3.85	0.054
Participation in physical activity, %						
Light activity	55.5	50.8	0.152	67.4	62.9	0.272
Moderate activity	27.4	20.6	0.017	15.1	7.3	0.009
Strenuous/muscle-conditioning activity	30.3	23.9	0.031	18.4	12.6	0.077
Lawn work/yard care/gardening	10.1	10.0	0.935	4.7	7.3	0.166
Light housework	80.7	73.4	0.006	93.1	89.4	0.101
Heavy housework	52.2	37.5	<0.0001	63.9	59.6	0.300
Home repairs	15.4	9.3	0.007	1.0	0.0	0.217
Caring for another person	15.2	10.3	0.029	12.6	10.6	0.474
Paid/volunteer work	19.4	16.6	0.279	11.9	11.9	0.981
Total energy intake, kcal	2147.64±582.93	2042.78±535.23	0.003	1609.41±467.43	1489.83±468.97	0.003
DQI	64.17±9.43	61.60±10.85	<0.0001	65.64±8.98	62.11±10.71	<0.0001
Frailty index	0.08±0.05	0.10±0.06	<0.0001	0.10±0.06	0.12±0.07	<0.0001

BMI, body mass index; DQI, dietary quality index.

Table 2 shows the crude and adjusted hazard ratio (HR) for all-cause mortality according to physical activity category. With the adjustment for age, education level, self-rated socioeconomic status, total energy intake, DQI, smoking and alcohol use, we found an inverse association between risk of all-cause mortality and heavy housework among men, with the adjusted HR of 0.72 (95%CI = 0.57–0.92). Further adjustment for BMI, frailty index, living arrangement, and level of leisure time activity did not change the result (HR = 0.71, 95%CI = 0.56–0.91). None of the physical activity measures bore any significant association with all-cause mortality among women.

**Table 2:** Separate Cox regression models linking physical activity to all-cause mortality in 2,867 Hong Kong elderly men and women.

		Men (n = 1,417)							Women (n = 1,450)						
Physical		Case/	Crude		Model 1^a^		Model 2^b^		Case/	Crude		Model 1^a^		Model 2^b^	
Activity		Control	HR	95%CI	HR	95%CI	HR	95%CI	Control	HR	95%CI	HR	95%CI	HR	95%CI
*Overall physical activity*															
1^st^ tertile		125/354	1	reference	1	reference	1	reference	54/427	1	reference	1	reference	1	reference
2^nd^ tertile		95/371	0.76	0.58–0.99	0.95	0.71–1.25	0.95	0.72–0.26	52/434	0.95	0.65–1.39	1.39	0.90–2.16	1.44	0.93–2.25
3^rd^ tertile		81/391	0.60	0.46–0.80	0.85	0.64–1.15	0.87	0.64–1.16	45/438	0.77	0.52–1.14	1.46	0.92–2.29	1.54	0.97–2.43
*Leisure time physical activity*															
Light	Inactive	148/497	1	reference	1	reference	1	reference	56/424	1	reference	1	reference	1	reference
	Active	153/619	0.84	0.67–1.06	0.92	0.73–1.17	0.94	0.74–1.20	95/875	0.85	0.61–1.18	0.91	0.63–1.32	0.89	0.61–1.30
Moderate	Inactive	239/810	1	reference	1	reference	1	reference	140/1,103	1	reference	1	reference	1	reference
	Active	62/306	0.71	0.53–0.93	0.84	0.63–1.12	0.88	0.66–1.17	11/196	0.43	0.23–0.79	0.56	0.30–1.05	0.56	0.30–1.05
Strenuous/muscle-conditioning	Inactive	229/778	1	reference	1	reference	1	reference	132/1,060	1	reference	1	reference	1	reference
	Active	72/338	0.74	0.57–0.97	0.96	0.73–1.27	1.00	0.76–1.32	19/239	0.65	0.40–1.05	0.85	0.51–1.40	0.85	0.52–1.41
Lawn work/yard care/gardening	No	271/1,003	1	reference	1	reference	1	reference	140/1,238	1	reference	1	reference	1	reference
	Yes	30/113	0.99	0.68–1.44	1.10	0.75–1.62	1.39	0.91–2.12	11/61	1.34	0.72–2.48	1.27	0.63–2.54	1.12	0.52–2.42
*Non-leisure time physical activity*															
Light housework	No	80/215	1	reference	1	reference	1	reference	16/90	1	reference	1	reference	1	reference
	Yes	221/901	0.68	0.53–0.88	0.84	0.64–1.09	0.82	0.63–1.08	135/1,209	0.64	0.38–1.07	0.82	0.47–1.45	0.83	0.47–1.48
Heavy housework	No	188/534	1	reference	1	reference	1	reference	61/469	1	reference	1	reference	1	reference
	Yes	113/582	0.58	0.46–0.74	0.72	0.57–0.92	0.71	0.56–0.91	90/830	0.84	0.60–1.16	1.11	0.77–1.59	1.13	0.77–1.65
Home repairs	No	273/944	1	reference	1	reference	1	reference	151/1,286	1	reference	1	reference	1	reference
	Yes	28/172	0.59	0.40–0.87	0.76	0.51–1.12	0.77	0.52–1.15	0/13	–	–	–	–	–	–
Caring for another person	No	270/946	1	reference	1	reference	1	reference	135/1,135	1	reference	1	reference	1	reference
	Yes	31/170	0.66	0.46–0.96	0.77	0.52–1.14	0.82	0.55–1.21	16/164	0.81	0.48–1.36	1.20	0.70–2.08	1.22	0.71–2.12
Paid/volunteer work	No	251/900	1	reference	1	reference	1	reference	133/1,145	1	reference	1	reference	1	reference
	Yes	50/216	0.85	0.63–1.15	1.17	0.86–1.60	1.23	0.90–1.69	18/154	1.02	0.62–1.67	1.03	0.61–1.74	1.05	0.61–1.79

a Mode1: Adjusted for age (continuous), education level (no education, primary, secondary/matriculation, and university or above), Hong Kong ladder (<5/≥5) total energy intake (continuous), DQI (continuous), smoking (never, past smoker, and current smoker), and alcohol use (never, past drinker, and current drinker).

b Model 2: Further adjusted for BMI (continuous), frailty index (continuous), living arrangement (live alone/live with spouse/others), and level of leisure time physical activity (continuous)/housework (continuous)*.

* Level of leisure time physical activity was adjusted in the models for the relationship between non-leisure time physical activity and all-cause mortality only while level of housework was adjusted in the models for the relationship between leisure time physical activity and all-cause mortality only.

Men: 1^st^ tertile, PASE <75.7143; 2^nd^ tertile, PASE 75.7143-<111.3333; 3^rd^ tertile, PASE ≥111.3333/Women: 1^st^ tertile, PASE <69.5714; 2^nd^ tertile PASE, 69.5714-<95.5476; 3^rd^ tertile 3, PASE ≥95.5476.

Table 3 shows the crude and adjusted HR for cancer mortality according to physical activity category. In men, heavy housework participation was associated with a significantly lower HR for cancer mortality after adjustment for age, education level, self-rated socioeconomic status, total energy intake, DQI, smoking, alcohol use, BMI, frailty index, living arrangement, and level of leisure time physical activity, with the HR = 0.52 (95%CI = 0.35–0.78). Men participated in light housework also were at lower risk of cancer mortality than were their counterparts, however, the association was not significant. None of the physical activity measures bore any significant association with cancer mortality among women.

**Table 3:** Separate Cox regression models linking physical activity to mortality due to cancer in 2,867 Hong Kong elderly men and women.

		Men (n = 1,417)							Women (n = 1,450)						
Physical		Case/	Crude		Model 1^a^		Model 2^b^		Case/	Crude		Model 1^a^		Model 2^b^	
Activity		Control	HR	95%CI	HR	95%CI	HR	95%CI	Control	HR	95%CI	HR	95%CI	HR	95%CI
*Overall physical activity*															
1^st^ tertile		49/354	1	reference	1	reference	1	reference	14/427	1	reference	1	reference	1	reference
2^nd^ tertile		40/371	0.79	0.52–1.20	0.87	0.57–1.34	0.87	0.57–1.35	26/434	1.78	0.93–3.42	1.92	0.94–3.96	1.99	0.97–4.10
3^rd^ tertile		34/391	0.62	0.40–0.97	0.84	0.53–1.33	0.84	0.53–1.33	22/438	1.43	0.73–2.80	1.82	0.86–3.84	1.94	0.91–4.12
*Leisure time physical activity*															
Light	Inactive	57/497	1	reference	1	reference	1	reference	27/424	1	reference	1	reference	1	reference
	Active	66/619	0.93	0.65–1.32	1.00	0.69–1.44	1.01	0.70–1.46	35/875	0.64	0.39–1.06	0.70	0.41–1.20	0.70	0.41–1.21
Moderate	Inactive	89/810	1	reference	1	reference	1	reference	58/1,103	1	reference	1	reference	1	reference
	Active	34/306	1.00	0.68–1.49	1.12	0.75–1.67	1.18	0.79–1.77	4/196	0.37	0.14–1.03	0.38	0.14–1.05	0.38	0.14–1.07
Strenuous/muscle-conditioning	Inactive	95/778	1	reference	1	reference	1	reference	50/1,060	1	reference	1	reference	1	reference
	Active	28/338	0.69	0.45–1.05	0.86	0.55–1.34	0.89	0.57–1.39	12/239	1.06	0.56–1.98	1.15	0.59–2.23	1.15	0.59–2.25
Lawn work/yard care/gardening	No	112/1,003	1	reference	1	reference	1	reference	58/1,238	1	reference	1	reference	1	reference
	Yes	11/113	0.90	0.48–1.67	1.07	0.57–2.06	1.52	0.77–3.02	4/61	1.24	0.45–3.43	1.21	0.43–3.43	0.93	0.29–2.95
*Non-leisure time physical activity*															
Light housework	No	36/215	1	reference	1	reference	1	reference	2/90	1	reference	1	reference	1	reference
	Yes	87/901	0.59	0.40–0.88	0.69	0.46–1.04	0.67	0.45–1.01	60/1,209	2.13	0.52–8.72	1.97	0.47–8.24	1.92	0.46–8.01
Heavy housework	No	82/534	1	reference	1	reference	1	reference	20/469	1	reference	1	reference	1	reference
	Yes	41/582	0.47	0.32–0.69	0.55	0.37–0.81	0.52	0.35–0.78	42/830	1.16	0.68–1.98	1.19	0.67–2.10	1.20	0.67−2.16
Home repairs	No	110/944	1	reference	1	reference	1	reference	62/1,286	1	reference	1	reference	1	reference
	Yes	13/172	0.65	0.37−1.16	0.77	0.43−1.37	0.75	0.42−1.35	0/13	–	–	–	–	–	–
Caring for another person	No	107/946	1	reference	1	reference	1	reference	53/1,135	1	reference	1	reference	1	reference
	Yes	16/170	0.83	0.49−1.40	0.98	0.56–1.63	0.96	0.56–1.64	9/164	1.15	0.57–2.33	1.27	0.61–2.66	1.33	0.63–2.80
Paid/volunteer work	No	98/900	1	reference	1	reference	1	reference	55/1,145	1	reference	1	reference	1	reference
	Yes	25/216	1.05	0.68–1.63	1.35	0.86–2.12	1.34	0.85–2.11	7/154	0.96	0.44–2.10	1.08	0.48–2.41	1.05	0.48–2.41

a Mode1: Adjusted for age (continuous), education level (no education, primary, secondary/matriculation, and university or above), Hong Kong ladder (<5/≥5) total energy intake (continuous), DQI (continuous), smoking (never, past smoker, and current smoker), and alcohol use (never, past drinker, and current drinker).

b Model 2: Further adjusted for BMI (continuous), frailty index (continuous), living arrangement (live alone/live with spouse/others), and level of leisure time physical activity (continuous)/housework (continuous)*.

* Level of leisure time physical activity was adjusted in the models for the relationship between non-leisure time physical activity and all-cause mortality only while level of housework was adjusted in the models for the relationship between leisure time physical activity and all-cause mortality only.

Men: 1^st^ tertile, PASE <75.7143; 2^nd^ tertile, PASE 75.7143-<111.3333; 3^rd^ tertile, PASE ≥111.3333/Women: 1^st^ tertile, PASE <69.5714; 2^nd^ tertile PASE, 69.5714-<95.5476; 3^rd^ tertile 3, PASE ≥95.5476.

## Discussion

We found that heavy housework participation was associated with reduced mortality and cancer deaths in older men over a 9-year period. This finding is in line with that of previous studies [13]-[17] which showed higher level of non-leisure time physical activity conferred survival benefits. However, light housework was not related to mortality. It is possible that associations might be apparent with only a wide variation in the level of participation but the majority (86%) in this study reported engaging in light housework. Mortality in women did not show any association with any of the non-leisure physical activity measures. The lack of associations in women might be explained by the small variability in energy expenditure, the small numbers of deaths during follow-up, or that mortality in older women might genuinely be independent of non-leisure time physical activity.

The underlying mechanism relating heavy housework participation to mortality needs further study. Most older men may not participate in leisure time activity of sufficient intensity to produce health benefits; heavy housework on the other hand may represent a certain level of energy expenditure that needs to be reached. For example, household activities such as vacuuming, mopping floors, and washing windows could contribute to the time spent in moderate intensity activity, which is defined as activity requiring an energy expenditure rate of 3.0–6.0 metabolic equivalents (METs) [26], [27]. Although our study did not estimate the energy expenditure on household activities, previous study revealed that energy expenditure of housework represented 35.2% of total activity in subjects aged 65–74 [11]. Indeed, many of the previous studies have focused on the health benefits of leisure time physical activity or recreational exercises, whereas calculating energy expenditure using only leisure time physical activity may underestimate the activity of many individuals whose activity was primarily housework [28]. Levine et al. also pointed out that the main component of total energy expenditure is non-exercise activity thermogenesis (NEAT), second only to basal metabolic rate and defined as the energy expenditure of all physical activities other that volitional sporting-like exercise [29]. As household activity comprises a substantial portion of total activity among older people [9]-[12], and as exercise and occupational activity generally decrease with ageing, household activity appeared to be a major determinant of NEAT such that elderly men who participated in heavy housework expended on more energy than those who did not participate, and were associated with reduced mortality. It has also been reported that NEAT can burn a tremendous amount of calories, which has been associated with reduced weight/obesity [30].

Another possible explanation for the significant association between heavy housework and mortality could be that men who do not do heavy housework are more likely to suffer from some illness or are more frail, i.e., heavy housework participation is a surrogate indicator of underlying health. To check for this possibility, subjects with diabetes, heart diseases, stroke or cancer at baseline were excluded in the analyses and BMI and frailty index were adjusted for. The results indicated that the beneficial impact of heavy housework remains significant. Also, despite the fact that housework participation was not correlated with leisure time physical activity, housework participation may be acting as proxy measures of physical activity. Therefore, leisure time physical activity was further adjusted in the model where heavy housework remained significant independent predictors of mortality and cancer deaths. Thus, this finding adds to our confidence that heavy housework participation exert an independent protective effect not due exclusively to the impact of leisure time physical activity.

The inverse relations between heavy housework and mortality in the present study may also be due to the enhanced psychosocial effects of performing housework. Previous studies suggested that productive activity such as gardening or preparing meals may result in a sense of meaning and purpose in life, which has been linked to survival [13]. Recent data also suggests that men are happier and have less psychological stress when they do the housework [31]. Therefore, non-leisure time physical activity including housework may have important health impacts that go beyond the benefits of fitness improvement. The underlying mechanism needs further study.

This finding has important implications for public health policy, suggesting that a broader range of physical activity that fits into everyday life, particularly household chores, should be promoted rather than placing emphasis on doing daily exercises. In the past, physical activity recommendations have mostly focused on physical activity in leisure time, yet physical inactivity remains a pressing public health issue. In Hong Kong, less than 30% of adults and the elderly did not meet current physical activity recommendations (accumulation of ≥150 minutes of moderate or above intensity physical activity in a week) [8] and in the present study, more than 75% of the subjects did not engage in sufficient physical activity. Promoting increases in household related activities might be of benefit, particularly among elderly people, as more people will be capable of achieving recommended levels of physical activity. In the present study, the prevalence of doing housework was 52–81% for males and 6–93% for females. Levels of participation in household activities found in the present study agree well with those reported in previous studies conducted in Europe for the elderly. In Nottingham, UK, indoor activities were the most frequently reported, 86% of the sample performing some indoor activities. 95% of the total time on indoor activities comprised housework [9]. In the British Women’s Heart and Health Study conducted in the UK, the prevalence of heavy housework (for at least 2.5 hrs per week) was 53% among women aged 60–79 [10]. Study conducted in Germany has also shown housework to be the most common physical activity among community-dwelling older adults aged 72–93, where 79% of men and 87% of women reported they had done heavy housework and/or gardening in the previous week [32]. As participating housework is a common practice of many families and is also socially acceptable, it is likely that intervention to promote housework participation could lead to substantial increase in level of physical activity with a cost saving to health sector.

In contrast to previous studies [2], we did not observed any association of overall physical activity, leisure time physical activities or other types of non-leisure time physical activities (such as home repairs, caring for others, paid/volunteer work) with mortality. The low levels of these activities in our sample may contribute to the non-significant results. Nevertheless, there are significant gender differences in participation in physical activity such as housework, where it is still more commonly carried out among women in Chinese society. Our findings revealed that women were less active overall but engaged more in both heavy and light housework than their male counterparts. Therefore, the health benefits of physical activity, particular daily housework participation should be promoted among both genders, which may provide insight into feasible and sustainable strategies that will increase total physical activity particular among those who are sedentary.

There are limitations in this study. Information on physical activity was self-reported, which invariable entails some degree of misclassification. The presence of a chronic disease was based on self-reports of a doctor’s diagnosis that were not verified, which may possibly result in some misclassification. Our cohort is more educated and more physically active than the general elderly population in Hong Kong; therefore, findings should not be generalized to those who are institutionalized or frailer, or with lower education level. The number of cancer deaths was small, especially among women; therefore, the power of analysis in demonstrating a genuine lack of relationship between physical activity and cancer deaths might be limited. A longer follow-up period with higher number of cancer deaths might be able to provide a more definitive answer.

In conclusion, heavy housework was associated with reduced mortality and cancer deaths in older men over a 9-year period. Efforts to promote non-leisure time physical activity, especially housework participation should be considered, which may increase levels of physical activity and confer survival benefits for elderly populations.
