# Correction: Housework Reduces All-Cause and Cancer Mortality in Chinese Men

**DOI:** 10.1371/annotation/0877cd36-268a-4ecd-af2e-fc24421c7bdc

**Published:** 2013-11-12

**Authors:** Ruby Yu, Jason Leung, Jean Woo

There was an percentage error for female subjects in the fifth paragraph in the discussion section. The corrected sentence is: In the present study, the prevalence of doing housework was 52–81% for males and 64–93% for females.

